# Hypocalcemia is associated with adverse outcomes in patients with diabetes and COVID-19

**DOI:** 10.3389/fendo.2025.1504326

**Published:** 2025-06-27

**Authors:** Xinyue Xu, Qin Zhu, Yaling Yang, Jia Liu, Chenwei Wu, Duoduo Qu, Chunhong Wang, Xiaolong Zhao

**Affiliations:** Department of Endocrinology, Shanghai Public Health Clinical Center, Shanghai, China

**Keywords:** COVID-19, hypocalcemia, diabetes, serum calcium, outcome

## Abstract

**Background:**

Patients with diabetes and COVID-19 have a worse prognosis. The aim of this study was to investigate the prevalence of hypocalcemia in patients with diabetes and COVID-19, and assess the relationship between serum calcium levels and prognosis in these patients.

**Methods:**

A retrospective analysis was conducted on 919 patients with diabetes admitted for COVID-19 from February 2022 to May 2022. The population was categorized into three groups according to serum calcium levels. The primary outcome was the risk of developing severe COVID-19, and the secondary outcomes included the risk of requiring advanced respiratory support (including high-flow oxygen, non-invasive ventilation, and invasive ventilation). Logistic regression analysis was used to evaluate the association between hypocalcemia and the prognosis of COVID-19 patients with diabetes.

**Result:**

Among the 919 patients with diabetes, the median age was 70 (56-81) years, and 498 (54.2%) were male. The prevalence of hypocalcemia in COVID-19 patients with diabetes was 78.8%. The serum calcium level was negatively correlated with inflammatory markers (hsCRP, ESR, PCT, IFN). The serum calcium level was positively correlated with albumin, CD4+ T cell counts, and CD8+ T cell counts. In Multivariate analysis, after adjustment for age and gender, the higher risk of severe illness was observed in patients with a serum calcium level <1.94 mmol/L (OR 2.86, 95%CI [1.78-4.59], P<0.001).

**Conclusion:**

Admission serum calcium level is associated with the prognosis of COVID-19 patients with diabetes. Hypocalcemia increases the risk of progression to severe COVID-19 in patients with diabetes.

## 1 Introduction

Severe Acute Respiratory Syndrome Coronavirus 2 is a type of coronavirus transmitted through respiratory droplets and contact, causing Coronavirus Disease 2019 (COVID-19) (1). COVID-19 is highly contagious and affects a broad population. With appropriate treatment, most patients exhibit a favorable prognosis. However, severe cases can rapidly advance to Acute Respiratory Distress Syndrome (ARDS), intractable metabolic acidosis, and coagulation dysfunction that is challenging to rectify. According to reports, approximately 10% of the COVID-19 population has diabetes (2, 3). Diabetes is one of the risk factors for severe COVID-19. Studies have shown that, compared to individuals without diabetes, patients with diabetes and COVID-19 tend to experience more severe clinical symptoms, and have worse biochemical indicators and outcomes (4). Diabetes impairs innate and humoral immunity. Patients with diabetes infected with COVID-19 have elevated levels of IL-6 and C-reactive protein, so the proinflammatory state of diabetes may favor the cytokine storm and systemic inflammatory response that accompanies ARDS in patients with COVID-19 (5).

COVID-19 can contribute to multisystem injury, severe COVID-19 patients may experience an imbalance of fluid and electrolyte owing to the stress response, of which hypocalcemia was one of the common clinical manifestations. Calcium ions function as crucial second messengers, intricately involved in the regulation of nearly all cellular processes within mammalian cells, and they play a fundamental role in the mechanism of viral replication (6, 7). Existing research has indicated a significant correlation between hypocalcemia and the prognosis of patients with COVID-19 pneumonia (8). Therefore, this study aims to investigate the prevalence of hypocalcemia in patients with diabetes and COVID-19 during the COVID-19 pandemic in Shanghai in the spring of 2022. Additionally, the study intends to analyze the relationship between serum calcium levels and the prognostic outcomes of these patients. This research could have significant implications for clinical practice by informing the management of calcium levels in patients with diabetes and COVID-19. Early identification and treatment of hypocalcemia in these patients may help mitigate the risk of severe outcomes, potentially leading to more targeted and effective treatment strategies.

## 2 Method

### 2.1 Research objects

The study was approved by the Ethics Committee of the Shanghai Public Health Clinical Centre (2023-S069-01). Patients with diabetes hospitalized with COVID-19 admitted to the Shanghai Public Health Clinical Center from February 1, 2022 to May 31, 2022 were enrolled for retrospective analysis. All patients were confirmed based on the “Diagnosis and Treatment Protocol for Novel Coronavirus Pneumonia (Trial 9th Edition)” published by the National Health Commission of China. The diagnostic criteria for severe case was that the patient meets any of the following conditions: (1) shortness of breath, respiratory rate ≥ 30 breaths/min; (2) Oxygen saturation ≤ 93% at rest while breathing ambient air; (3) Arterial oxygen partial pressure (PaO_2_)/fraction of inspired oxygen (FiO_2_) ≤ 300 mmHg; (4) Progressive worsening of clinical symptoms, with imaging showing significant progression of lung lesions >50% within 24–48 hours. The diagnostic criteria for critical case was that the patient meets any of the following conditions: (1) respiratory failure requiring mechanical ventilation; (2) onset of shock; (3) multiple organ dysfunction requiring monitoring and treatment in the Intensive Care Unit. Severe and critical patients were categorized as severe cases. Inclusion criteria: 1) diagnosis of COVID-19 confirmed by PCR from nasal or oral swabs; 2) for patients with diabetes, the diagnosis is based on medical history or hemoglobin A1c (HbA1c) level ≥ 6.5% measured after admission. Exclusion criteria: (1) age under 18 years; (2) estimated glomerular filtration rate(eGFR) < 15 ml/min/1.73m^2^; (3) incomplete medical records.

### 2.2 Data collection and research outcomes

Data on gender, age, medical history, vaccination status, complications, and outcomes, were collected from all subjects. Within 24 hours of admission, routine biochemical parameters, inflammatory markers, immune cell counts, and electrolyte metabolism parameters were collected. Hypocalcemia was defined as serum calcium levels <2.2 mmol/L (9). Albumin adjusted calcium (mmol/L) = total calcium (mmol/L) + 0.02 [40 – albumin (g/L)]. The study population was divided into tertiles categories according to the serum calcium level on admission, serum calcium < 1.94 mmol/L in group low, ≥ 1.94 mmol/L and ≤ 2.11 mmol/L in group medium; serum calcium >2.11 mmol/L in group high. eGFR was calculated using the CKD Epidemiology Collaboration (CKD-EPI) Creatinine Equation. The primary outcome was the development of severe COVID-19, and the secondary outcome was the need for advanced respiratory support therapy (including high-flow oxygen, non-invasive ventilation, and invasive ventilation).

### 2.3 Data analysis

Normality was tested using the shapiro-wilk test, and p < 0.05 was considered as not obeying a normal distribution. Normally distributed data are expressed as means ± SD and independent samples t-test was used to compare between the 2 groups. Non-normally distributed data are expressed as median (interquartile range), and comparisons between groups were performed using the Mann-Whitney U test. Spearman correlation analysis was used to evaluate the correlation between various indicators. Logistic regression was used to analyze the association between hypocalcemia and the prognosis of patients with COVID-19 and diabetes. P < 0.05 was considered as a statistically significant difference. SPSS 19.0 software was used for statistical analysis.

## 3 Results

### 3.1 Baseline data of the overall study population.

**Table 1** shows the main clinical characteristics of the study population. 919 people were included in this study, aged 70 (56-81) years, and 498 (54%) were male. In this cohort, 10.4% of patients progressed to severe COVID-19, 9.1% of patients needed advanced respiratory support therapy, HbA1c was 6.9 (6.6-8.2) %, serum calcium level was 2.01 (1.91-2.17) mmol/L, and 78.8% of patients developed hypocalcemia. The proportion of severe COVID-19 cases in group low (20.5% vs 5.5% vs 3.5%), the proportion of requiring advanced respiratory support therapy (18.6% vs 5.2% vs 4.5%), and the number of days of hospitalization (9 [6-15] vs 9 [5-13] vs 11 [6-14]) were significantly more than those in groups medium and high. p< 0.05. Among them, patients in group medium (6.9 [6.6-8.0]%) had better HbA1c than group low (6.9 [6.5-8.1]%) and group high (7 [6.6-8.8]%), P < 0.05. The CD4+ T cell counts and CD8+ T cell counts in group low were significantly lower than those in group medium, P < 0.05. Interleukin-6 (IL-6) in group low was significantly higher than those in group medium and group high, P < 0.05.

**Table 1:** Main clinical features of the study group by tertile categories of serum calcium at hospital admission.

Parameter	Total (n=919)	Group I (<1.94, n=306)	Group II (1.94~2.11, n=306)	Group III (>2.11, n=307)
Male (%)	498 (54.2%)	176 (57.5%)	155 (50.7%)	167 (54.4%)
Severe (%)	96 (10.4%)	64 (17.6%)^ab^	19 (6.2%)^c^	13 (4.2%)
Vaccination (%)	288 (31.3%)	45 (14.7%)^ab^	91 (29.7%)^c^	152 (49.5%)
ARST (%)	84 (9.1%)	57 (18.6%)^ab^	13 (4.2%)^c^	14 (4.6%)
Age (years)	70 (56-81)	77 (68-87) ^ab^	69 (58-79) ^c^	60 (47.5-74)
Length of Hospitalisation (days)	10 (5-14)	9 (5-15) ^a^	9 (5-13) ^c^	11 (5-14)
HbA1c (%)	6.9 (6.6-8.2)	6.9 (6.5-8.1) ^ab^	6.9 (6.6-8.0) ^c^	7 (6.6-8.8)
Serum Calcium (mmol/L)	2.01 (1.91-2.17)	1.86 (1.8-1.91) ^ab^	2 (1.97-2.06) ^c^	2.22 (2.17-2.30)
Albumin adjusted calcium (mmol/L)	2.03 (1.93-2.18)	1.89 (1.82-1.97) ^ab^	2.01 (1.95-2.07) ^c^	2.22 (2.15-2.34)
Phosphorus (mmol/L)	0.86 (0.53-1.14)	0.9 (0.54-1.15)	0.95 (0.53-1.15)^c^	0.68 (0.51-1.13)
Sodium (mmol/L)	139 (137-141)	138 (135-141)^b^	139 (137-140)	139 (137-141)
ALT (U/L)	19 (12-30.8)	17 (12-29)	20 (13-29.8)	19 (12.5-32)
AST (U/L)	20 (16-29)	21 (16-30)	20 (17-27)	20 (15-28)
LDH (U/L)	203 (176-243)	211 (176-251) ^ab^	203 (177-236)	199 (175-234)
Albumin (g/dL)	40 (36-42)	38 (34.5 -41) ^ab^	40 (37.8 -43)	40 (36-43)
Globulin (g/dL)	29 (26-32)	29 (27-33)	29 (26-31)	29 (26-32)
Urea (mmol/L)	5.4 (4.2-6.9)	7 (5.6-9.6) ^ab^	5.1 (4.2-6.1) ^c^	4.6 (3.6-5.8)
UA (umol/L)	296.5 (239.5-363.9)	349.0 (277.8-418.5) ^ab^	289.5 (241.1-347.9) ^c^	269 (211.8-327.4)
eGFR (ml/min/1.73m^2)^	95.0 (75.4-114.5)	66.7 (54.3-74.1) ^ab^	94.0 (87.5-100.2) ^c^	123.4 (113.8-139.4)
hsCRP (mg/L)	2.77 (0.50-13.63)	4.15 (0.78-18.29) ^ab^	2.12 (0.50-6.52)	2.62 (0.50-16.72)
CD4 cell counts (cell/uL)	583.74 (394.83-808.78)	525.75 (359.34-779.79)^a^	633.14 (436.27-865.87)	576.6 (393.31-782.81)
CD8 cell counts (cell/uL)	327.96 (210.77-497.28)	301.77 (190.21-458.05) ^ab^	333.18 (234.78-530.21)	334.10 (204.60-520.05)
ESR (mm/h)	28 (10-68)	36 (14-74) ^ab^	26 (9-67)	23 (9-54)
PCT (ng/mL)	0.1 (0.1-0.1)	0.1 (0.1-0.14) ^ab^	0.1 (0.1-0.1)	0.1 (0.1-0.1)
IFN-a (pg/mL)	0.81 (0.38-1.79)	0.78 (0.36-2.04)	0.82 (0.37-1.68)	0.81 (0.40-1.87)
IL-5 (pg/mL)	1.58 (1.08-2.46)	1.36 (0.98-2.37)^a^	1.63 (1.19-2.39)	1.65 (1.90-2.52)
IL-6 (pg/mL)	2.13 (1.13-4.84)	2.57 (1.23-6.76) ^ab^	1.96 (1.15-3.97)	1.98 (1-4.22)

Continuous variables are expressed as median (interquartile range). Discrete traits are expressed as n (%) Abbreviations as described in the text.

ARST, advanced respiratory support therapy.

a< 0.05 group I vs group II.

b < 0.05 group I vs group III.

c < 0.05 group II vs group III.

### 3.2 The level of serum calcium was closely related to other inflammatory factors and cellular immune function.

**Table 2** shows that the serum calcium level was negatively correlated with age, phosphorus, lactate dehydrogenase (LDH), uric acid (UA), urea, high-sensitivity CRP (hsCRP), erythrocyte sedimentation rate (ESR), procalcitonin (PCT), and interferon-a (IFN-a). The serum calcium level was positively correlated with albumin, eGFR, CD4+ T cell counts and CD8+ T cell counts.

**Table 2:** Correlations of calcium with other indicators in patients.

Parameter	R	P value
Age (years)	-0.352	<0.001
Length of Hospitalisation(days)	-0.044	0.178
HbA1c (%)	0.041	0.224
Phosphorus (mmol/L)	-0.069	0.036
Sodium (mmol/L)	0.110	0.001
ALT(U/L)	-0.006	0.851
AST(U/L)	-0.086	0.009
LDH (U/L)	-0.106	0.001
Albumin (g/dL)	0.112	0.001
Globulin (g/dL)	-0.046	0.162
Urea (mmol/L)	-0.479	<0.001
Uric acid (umol/L)	-0.399	<0.001
eGFR (mL/min/1.73m^2)^	0.949	<0.001
hsCRP (mg/L)	-0.085	0.011
CD4 cell counts (cell/uL)	0.091	0.006
CD8 cell counts (cell/uL)	0.122	<0.001
ESR (mm/h)	-0.091	0.008
PCT (ng/mL)	-0.081	0.015
IFN-a (pg/mL)	-0.088	0.01
IL-5 (pg/mL)	-0.032	0.346
IL-6 (pg/mL)	-0.065	0.058

### 3.3 Lower serum calcium level was related to adverse outcome

As shown in **Table 3**, we analyzed the factors influencing the adverse outcome of COVID-19 in patients with diabetes. In univariate analysis, several factors, including advanced age, high aspartate transaminase (AST) level, high hsCRP level, high ESR level, and hypocalcemia, were identified as potential risk factors for adverse outcomes in patients with COVID-19 and diabetes. In Multivariate analysis, after adjustment for age and gender, the higher risk of severe illness was observed in patients with a serum calcium level < 1.94 mmol/L (OR 2.86, 95%CI [1.78-4.59], P < 0.001). Vaccination reduced the risk of patients progressing to severe COVID-19 (OR 0.22, 95%CI [0.09-0.57], P < 0.001.

**Table 3:** Univariate and multivariate analysis of the progression to severe cases for all individuals.

Parameter		Univariate, OR (95%CI)	P value	Multivariate, OR (95%CI)^a^	P value
Age (years)		1.07 (1.05-1.09)	<0.001		
Gender (Male)		1.15 (0.75-1.76)	0.519		
Vaccination (%)		0.1 (0.04-0.26)	<0.001	0.22 (0.09-0.57)	0.002
Serum Calcium (mmol/L)		0.02 (0.00-0.06)	<0.001	0.06 (0.02-0.23)	<0.001
Grouping of serum calcium	group II-III	1 (ref)			
	group I	4.8 (3.06-7.53)	<0.001	2.86 (1.78-4.59)	<0.001
HbA1c (%)		0.95 (0.83-1.07)	0.388		
ALT (U/L)		1.00 (1.00-1.09)	0.314		
AST (U/L)		1.01 (1.01-1.01)	0.012	1.01 (1.01-1.01)	0.05
Albumin (g/dL)		0.95 (0.91-0.98)	0.003	0.95 (0.91-0.99)	0.01
eGFR (ml/min/1.73m^2)^		0.97 (0.99-0.99)	<0.001	0.98 (0.97-0.99)	<0.001
hsCRP (mg/L)		1.01 (1.01-1.01)	0.016	1.01 (1.01-1.01)	0.024
CD4 cell counts (cell/uL)		0.99 (0.99-0.99)	0.004	0.99 (0.99-0.99)	0.032
CD8 cell counts (cell/uL)		1.00 (1.00-1.00)	0.247		
ESR (mm/h)		1.01 (1.01-1.01)	0.009	1.01 (1.01-1.01)	0.031
PCT (ng/mL)		1.01 (0.92-1.11)	0.876		
IFN-a (pg/mL)		1.00 (0.94-1.06)	0.905		
IL-5 (pg/mL)		1.00 (0.96-1.04)	0.982		
IL-6 (pg/mL)		1.00 (1.00-1.01)	0.274		

a Adjusted for age and gender.

## 4 Discussion

In this retrospective cohort study involving 919 patients with diabetes and COVID-19, we observed a high prevalence of hypocalcemia in hospitalized patients. Patients with hypocalcemia generally had worse clinical outcomes, with a significantly increased risk of developing severe COVID-19 and requiring advanced respiratory support therapy. Our findings align with previous research by Di Filippo et al., which also reported a high prevalence of hypocalcemia in COVID-19 patients, particularly those with severe disease (8). Their study noted that over 70% of COVID-19 patients exhibited hypocalcemia upon admission, consistent with our observed rate of 78.8% in diabetic patients (8). This reinforces the notion that hypocalcemia is a common and significant feature in COVID-19, especially among those with comorbid conditions like diabetes.

The relationship between hypocalcemia and COVID-19 prognosis is complex. Our study demonstrates a significant association between low serum calcium levels and severe outcomes in diabetic COVID-19 patients. However, it remains unclear whether hypocalcemia is a causative factor for disease progression or merely a marker of severe illness. Recent evidence suggests that COVID-19 may have a direct role in disrupting calcium metabolism, potentially contributing to hypocalcemia (10). COVID-19 may affect calcium homeostasis through mechanisms such as viral entry into host cells and the hyper-inflammatory state seen in critically ill patients. The cytokine storm associated with severe COVID-19 can lead to increased systemic inflammation, multi-organ dysfunction, and secondary hypocalcemia. Some studies, like the one by Mardani et al., suggest that hypocalcemia may be a marker of severe inflammation rather than a direct causative factor for disease progression (11). They argue that hypocalcemia reflects the extent of systemic inflammation and multi-organ involvement, which are prevalent in critically ill patients. This view contrasts with our findings, where hypocalcemia appears to play a more active role in worsening clinical outcomes. These differences may be attributed to variations in study design, patient populations, and the timing of serum calcium assessments.

Vitamin D can regulate serum calcium levels and has immunomodulatory functions (12). Previous studies have shown that vitamin D levels were associated with the severity and mortality of COVID-19 (13). Hypocalcemia has been associated with poor prognosis in COVID-19, which may be due to a deficiency in vitamin D. Hypovitaminosis D not only disrupts calcium homeostasis but also plays a crucial role in immune function. Emerging evidence suggests that hypovitaminosis D may impair the long-term immune response following vaccination, potentially leading to suboptimal vaccine efficacy (14). In addition, hypovitaminosis D is recognized as a significant risk factor for the development of diabetes itself, further complicating the clinical scenario in patients with diabetes and COVID-19 (15, 16). Therefore, vitamin D deficiency not only contributes to hypocalcemia but also worsens the overall prognosis by increasing the risk of metabolic disorders such as diabetes, which are known to exacerbate COVID-19 outcomes. By influencing both calcium metabolism and immune responses, vitamin D status may play a critical role in determining the clinical course of COVID-19 in patients with diabetes.

In this study, the eGFR level was significantly lower in the lowest serum calcium group compared to the other two groups. In addition, we observed a clear correlation between serum calcium levels and other biochemical indicators such as phosphorus and uric acid, which may indicate that cell damage and metabolic disorders are exacerbated in severe COVID-19 cases. Patients with diabetes were at increased risk of kidney failure, and in the event of kidney failure, phosphorus and uric acid cannot be effectively excreted from the body, resulting in hyperphosphatemia and hyperuricemia. Hyperphosphatemia induces hypocalcemia by interfering with phosphorus excretion in the dysfunctional kidneys (17, 18). In addition, phosphate binds to ionized calcium and removes it from the serum. Severe COVID-19 patients secrete a large amount of inflammatory factors, which changes the permeability of the cell membrane, causing calcium influx and reducing the activity of calcium pumps, resulting in a decrease in serum calcium (19). Severe pulmonary patients have many complications, often combined with gastrointestinal diseases, liver and kidney dysfunction, and reduced calcium intake, absorption, and synthesis (20). Patients with COVID-19 were prone to hypoalbuminemia (especially albumin). Serum albumin is highly positively correlated with serum calcium concentration, causing patients to reduce both bound calcium and ionized calcium (21). Severe COVID-19 patients are characterized by a hyperinflammatory response with high levels of inflammatory and organ injury biomarkers. The serum calcium level was negatively correlated with several inflammatory markers (hsCRP, RSR, PCT, IFN-a, AST, LDH), suggesting that an inflammatory response was present early on in COVID-19 patients in the lowest calcium group and that an “inflammatory factor storm” may have occurred, resulting in the progression of the patient to severe disease. Hypocalcemia can also cause various physiological function problems such as nerve conduction, muscle contraction and tension, hormone synthesis and secretion, etc. Patients may experience abnormal sensations, numbness of limbs, and in severe cases, convulsions, spasms, QT interval prolongation, etc. For patients who require advanced respiratory support, especially mechanical ventilation, severe hypocalcemia may cause laryngospasm and cardiac dysfunction, which will prolong the patient’s mechanical ventilation time. If not treated in time, it may increase the patient’s risk of death (22).

It is noteworthy that our findings indicate that patients with a lower proportion of vaccination had the most severe hypocalcemia. This association requires further investigation to elucidate the underlying mechanisms. It is plausible that unvaccinated patients, who are at an elevated risk of severe illness, may experience more pronounced systemic inflammation and organ dysfunction, leading to greater disturbances in calcium homeostasis. Vaccination, by reducing the severity of COVID-19 infection, could mitigate these effects. Further studies are required to explore the exact relationship between vaccination status and hypocalcemia in patients with COVID-19 infection.

Due to Chinese specific treatment protocols for COVID-19 in the past, all patients with COVID-19 required hospitalization, and the sample of people included in this study is relatively more comprehensive. Consequently, the population examined in this study is relatively comprehensive, allowing for a focused investigation into electrolyte imbalances occurring in diabetic patients after COVID-19 infection. 919 people were included in this study. Hypocalcemia plays a significant role in adverse outcomes in COVID-19 patients with diabetes, and the article outlines potential causes and mechanisms influencing calcium levels.

However, the study has some limitations: 1. the study lacked crucial parameters, such as 1,25(OH)2D3 and PTH levels, which play a key role in calcium regulation; 2. this is a retrospective study and cannot establish a causal relationship; 3. This study did not analyze the effect of smoking on the prognosis of patients with new crowns, and some studies have shown that smoking may affect the prognosis of patients with COVID-19 (23).

## 5 Conclusions

In summary, patients with COVID-19 and diabetes are prone to hypocalcemia, the serum calcium level was negatively correlated with inflammatory factors. Patients with serum < 1.94 mmol/L were approximately 2.8-fold increased probability of progressing to severe COVID-19 than other patients. Hypocalcemia was associated with adverse outcomes in patients with COVID-19 and diabetes.

## Data Availability

The data analyzed in this study is subject to the following licenses/restrictions: The datasets used and/or analyzed during the current study are available from the corresponding author on reasonable request. Requests to access these datasets should be directed to xiaolongzhao@163.com.

## References

[1] Wu F Zhao S Yu B Chen YM Wang W Song ZG. A new coronavirus associated with human respiratory disease in China. Nature. (2020) 579:265–9. doi: 10.1038/s41586-020-2008-3 PMC7094943 32015508

[2] Chen N Zhou M Dong X Qu J Gong F Han Y. Epidemiological and clinical characteristics of 99 cases of 2019 novel coronavirus pneumonia in Wuhan, China: a descriptive study. Lancet. (2020) 395:507–13. doi: 10.1016/s0140-6736(20)30211-7 PMC7135076 32007143

[3] Wang D Hu B Hu C Zhu F Liu X Zhang J. Clinical characteristics of 138 hospitalized patients with 2019 novel coronavirus-infected pneumonia in wuhan, China. Jama. (2020) 323:1061–9. doi: 10.1001/jama.2020.1585 PMC7042881 32031570

[4] Seiglie J Platt J Cromer SJ Bunda B Foulkes AS Bassett IV. Diabetes as a risk factor for poor early outcomes in patients hospitalized with COVID-19. Diabetes Care. (2020) 43:2938–44. doi: 10.2337/dc20-1506 PMC7770271 32847827

[5] Lima-Martínez MM Carrera Boada C Madera-Silva MD Marín W Contreras M. COVID-19 and diabetes: A bidirectional relationship. Clin Investig Arterioscler. (2021) 33:151–7. doi: 10.1016/j.arteri.2020.10.001 PMC7598432 33303218

[6] Brown EM. Role of the calcium-sensing receptor in extracellular calcium homeostasis. Best Pract Res Clin Endocrinol Metab. (2013) 27:333–43. doi: 10.1016/j.beem.2013.02.006 23856263

[7] Zhou Y Frey T K Yang JJ. Viral calciomics: interplays between Ca2+ and virus. Cell Calcium. (2009) 46:1–17. doi: 10.1016/j.ceca.2009.05.005 19535138 PMC3449087

[8] Minasi A Andreadi A Maiorino A Giudice L De Taddeo S D'Ippolito I. Hypocalcemia is associated with adverse outcomes in patients hospitalized with COVID-19. Endocrine. (2023) 79:577–86. doi: 10.1007/s12020-022-03239-w PMC9643940 36350462

[9] di Filippo L Allora A Locatelli M Rovere Querini P Frara S Banfi G. Hypocalcemia in COVID-19 is associated with low vitamin D levels and impaired compensatory PTH response. Endocrine. (2021) 74:219–25. doi: 10.1007/s12020-021-02882-z PMC8480127 34586582

[10] di Filippo L Doga M Frara S Giustina A. Hypocalcemia in COVID-19: Prevalence, clinical significance and therapeutic implications. Rev Endocr Metab Disord. (2022) 23:299–308. doi: 10.1007/s11154-021-09655-z 33846867 PMC8041474

[11] Izzi-Engbeaya C Distaso W Amin A Yang W Idowu O Kenkre JS. Adverse outcomes in COVID-19 and diabetes: a retrospective cohort study from three London teaching hospitals. BMJ Open Diabetes Res Care. (2021) 9(1):e001858. doi: 10.1136/bmjdrc-2020-001858 PMC7789097 33408084

[12] Fleet JC. Vitamin D-mediated regulation of intestinal calcium absorption. Nutrients. (2022) 14(16):3351. doi: 10.3390/nu14163351 36014856 PMC9416674

[13] Jamilian A Ghalichi F Hamedi Kalajahi F Radkhah N Jourabchi N Musazadeh V. The role of vitamin D in outcomes of critical care in COVID-19 patients: Evidence from an umbrella meta-analysis of interventional and observational studies. Public Health Nutr. (2024) 27:1–25. doi: 10.1017/s1368980024000934 PMC11112434 38654693

[14] di Filippo L Frara S Terenzi U Nannipieri F Locatelli M Ciceri F. Lack of vitamin D predicts impaired long-term immune response to COVID-19 vaccination. Endocrine. (2023) 82:536–41. doi: 10.1007/s12020-023-03481-w PMC10618322 37592162

[15] Giustina A Bilezikian JP Adler RA Banfi G Bikle DD Binkley NC. Consensus statement on vitamin D status assessment and supplementation: whys, whens, and hows. Endocr Rev. (2024) 45:625–54. doi: 10.1210/endrev/bnae009 PMC11405507 38676447

[16] di Filippo L Giustina A. Vitamin D deficiency and type 2 diabetes: the dangerous link between two modern pandemics. J Clin Endocrinol Metab. (2024) 110:e905–6. doi: 10.1210/clinem/dgae390 38870277

[17] Blaine J Chonchol M Levi M. Renal control of calcium, phosphate, and magnesium homeostasis. Clin J Am Soc Nephrol. (2015) 10:1257–72. doi: 10.2215/cjn.09750913 PMC4491294 25287933

[18] Liamis G Liberopoulos E Barkas F Elisaf M. Diabetes mellitus and electrolyte disorders. World J Clin cases. (2014) 2:488–96. doi: 10.12998/wjcc.v2.i10.488 PMC4198400 25325058

[19] Hansen B A Bruserud Ø. Hypomagnesemia in critically ill patients. J Intensive Care. (2018) 6:21. doi: 10.1186/s40560-018-0291-y 29610664 PMC5872533

[20] Fernández R Cortés P Del Rio R Acuña-Castillo C Reyes EP. Lipopolysaccharide-induced ionized hypocalcemia and acute kidney injury in carotid chemo/baro-denervated rats. Adv Exp Med Biol. (2015) 860:161–6. doi: 10.1007/978-3-319-18440-1_18 26303478

[21] Sanaie S Mahmoodpoor A Hamishehkar H Shadvar K Salimi N Montazer M. Association between disease severity and calcium concentration in critically ill patients admitted to intensive care unit. Anesth Pain Med. (2018) 8:e57583. doi: 10.5812/aapm.57583 29868455 PMC5970362

[22] Iqbal M Rehmani R Hijazi M Abdulaziz A Kashif S. Hypocalcemia in a Saudi intensive care unit. Ann Thorac Med. (2008) 3:57–9. doi: 10.4103/1817-1737.39638 PMC2700460 19561907

[23] van Zyl-Smit RN Richards G Leone FT. Tobacco smoking and COVID-19 infection. Lancet Respir Med. (2020) 8:664–5. doi: 10.1016/s2213-2600(20)30239-3 PMC7247798 32464099

